# Protein Precipitation by Metal Hydroxides as a Convenient and Alternative Sample Preparation Procedure for Bioanalysis

**DOI:** 10.3390/molecules30010002

**Published:** 2024-12-24

**Authors:** Emanuele Salina, Luca Regazzoni

**Affiliations:** Department of Pharmaceutical Sciences, University of Milan, Via Mangiagalli 25, 20133 Milan, Italy

**Keywords:** protein precipitation, metal hydroxides, liquid chromatography, mass spectrometry, UV spectroscopy

## Abstract

Protein precipitation is widely used for sample preparation ahead of liquid chromatography. This step is required to analyze small molecules without the interference of proteins contained in the matrix. Organic solvents and acidic chemicals are the two most popular reagents used for this scope. Organic solvents are quite effective precipitating agents, but require a medium-to-large sample dilution. Moreover, a high concentration of organic solvents in sample media can affect reversed phase separations. Therefore, an evaporation step, followed by the resuspension of the analytes in appropriate media, is sometimes required. On the contrary, the addition of acidic compounds is more straightforward, since it keeps the supernatant aqueous and does not require evaporation, but the extreme pH can cause the degradation of analytes and the stationary phase. Herein, an alternative method for protein precipitation using the addition of zinc hydroxide was tested. The main advantages of this method over the other precipitating reagents are the minimal sample dilution required and the maintenance of aqueous media at nearly neutral pH which ensure analyte stability. The protocol ensured an effective protein removal before the analysis of small molecules in biological matrices, resulting in full compatibility with reversed phase chromatography coupled with both UV and mass spectrometric detectors.

## 1. Introduction

Bioanalysis refers to the identification and quantitation of analytes in biological matrices [1]. When the goal is protein characterization (e.g., proteomics studies, antibody purification), the isolation, purification, or extraction of proteins is required to remove small molecular weight interferents (e.g., salts, detergents). This can be conveniently achieved by inducing precipitation of the protein of interest, followed by washing steps and protein re-solubilization [2,3]. Protein precipitation can be achieved through thermal shock or by altering the solvent pH, ionic strength, and polarity through the addition of specific chemicals [2]. The use of chemicals is far more popular, since heating can cause irreversible denaturation that makes the redissolution of the proteins impossible [4]. The most popular reagents used for the purification, extraction, or isolation of specific proteins are trichloroacetic acid (TCA) and ammonium sulfate. Such reagents induce protein precipitation through different mechanisms that have been extensively studied [5,6,7]. Other reagents such as polyethylene glycol (PEG) and polyethyleneimine have been reported as well, although their use is less popular and the mechanisms inducing protein precipitation are not fully characterized [3,8,9].

Protein precipitation is also very useful for removing proteins interfering with the analyses of small molecules (e.g., metabolomics studies, pharmacokinetics studies on small molecules), especially ahead of chromatographic methods. Several studies have been published where the most popular reagents reported in the literature have been compared in terms of their efficacy for removing interfering proteins, without altering the performances of chromatographic methods [10,11,12,13]. Since the chemicals used to precipitate proteins alter the solvent pH, ionic strength, or polarity, there is no optimal protocol and any method can undergo a low analyte recovery [13,14]. Furthermore, the protein precipitation protocol must be carefully selected in view of the analytical methods used for sample analysis. For instance, precipitation with organic solvents can affect methods based on reversed phase chromatography, especially when the analytes of interest are polar compounds that have a short retention time [15]. To overcome such an issue, a further dilution to reduce organic solvent content can be used [16]. Alternatively, an evaporation step followed by redissolution is often required after protein precipitation [15]. Despite such a procedure requiring additional steps during sample preparation, the advantage is that samples can be concentrated. However, this increases the risks of poor analyte recovery due to thermal instability, adsorption, or poor solubility of analytes after solvent evaporation [14,17]. Moreover, since the evaporation followed by resuspension can undergo volumetric errors, it is often required to add an internal standard to the sample [15]. Nevertheless, there are also chromatographic methods not affected by such issues, and even reversed phase separation is often compatible with the injection of samples containing a high percentage of organic solvent [18,19,20]. Acetonitrile is the preferred solvent for many applications, since it performs a better removal of protein and other interferents compared to ethanol or methanol [10,12]. As reported for protein precipitation based on methanol, the interferents that are not removed can cause analytical issues during both chromatographic separation and electrospray ionization, which is a fundamental ionization technique for methods based on mass spectrometry detection (LC-ESI-MS) [15,16]. However, acetonitrile might not be the best choice for the analysis of very lipophilic compounds. In fact, there are some studies showing that other solvents such as isopropanol or butanol overperform acetonitrile for lipidomics [21,22]. The most popular alternative to organic solvents is the use of acidic compounds such as trichloroacetic acid (TCA), perchloric acid (PCA) and sulfosalicylic acid (SSA). The advantage of using such reagents is the minimal sample dilution required to precipitate proteins [12,17]. However, the low pH of supernatants may not be compatible with hydrolysable analytes as recently reported for the analysis of beta lactam antibiotics [17].

Precipitation protocols based on saturated ammonium sulfate are instead not so popular as a sample preparation method for chromatography. The main reason for this is that ionization suppression can occur when the matrix contains a high concentration of acidic compounds or salts [23,24]. In this context, the effects of TCA, SSA, and PCA on ionization are debated. Some authors suggested that such compounds can induce significative ionization suppression, while others advocate full compatibility with LC-ESI-MS [10,13,17,25,26]. However, ionization suppression can also be induced by acetonitrile, so it can also be an issue when protein precipitation is performed by diluting the sample in organic solvents [27]. The negative impact on ionization is more significant at the beginning of the chromatographic separation. Therefore, when reversed phase chromatography is used ahead of mass spectrometry, it is more impacting for the analysis of polar compounds [10,13].

Another issue with the use of acidic agents for protein precipitation is the chemical compatibility with the hardware of chromatographic instruments. Specifically, corrosion of the injection system was detected upon the injection of several samples treated with PCA. The analyses were performed according to a published method for the quantification of anthranilic acid (3HAA) in homogenates of mice brains, which required protein precipitation by means of PCA [28]. Precipitation with acetonitrile was tested, but it was not a suitable alternative since an efficient protein precipitation requires a 1–10 sample dilution, which is not compatible with the sensitivity of the method when a UV detector was used instead of MS. Furthermore, the retention of 3HAA was affected by the injection of samples containing high percentages of acetonitrile or other organic solvents. This was the starting point for the optimization of an alternative protein precipitation protocol, the details of which are reported in the Results section. Alternative reagents for protein precipitation were screened among the protocols reported in the literature to provide compatibility with the quantification of 3HAA in the homogenates of mice brains [28]. The tests were performed looking at chemicals not interfering with UV or mass spectrometric detection and requiring a minimal sample dilution to preserve method sensitivity, especially when UV was used as detector. Other features that were considered fundamental were the maintenance of a polar media in the samples to ensure no interference with retention of polar analytes such as 3HAA, as well as the maintenance of a pH in a range that avoids potential analyte hydrolysis or corrosion of instrument hardware (e.g., injection system, column). Three potential reagents were identified based on the literature data, namely ammonium sulfate (saturated aqueous solution), PEG 6000 (50% *w*/*v*), and a mixture of zinc sulfate and sodium hydroxide [8,9,10,12]. Other reagents were discarded since they were not compliant with the desired features reported above. Specifically, metaphosphoric acid was not considered since previous studies gave low and inconsistent protein precipitation across samples of plasma from different species [10], whereas sulfosalicylic acid was not considered because of its strong acidity (pKa = −0.62) and potential interference with UV detection [29,30].

## 2. Results and Discussion

**Figure 1:**
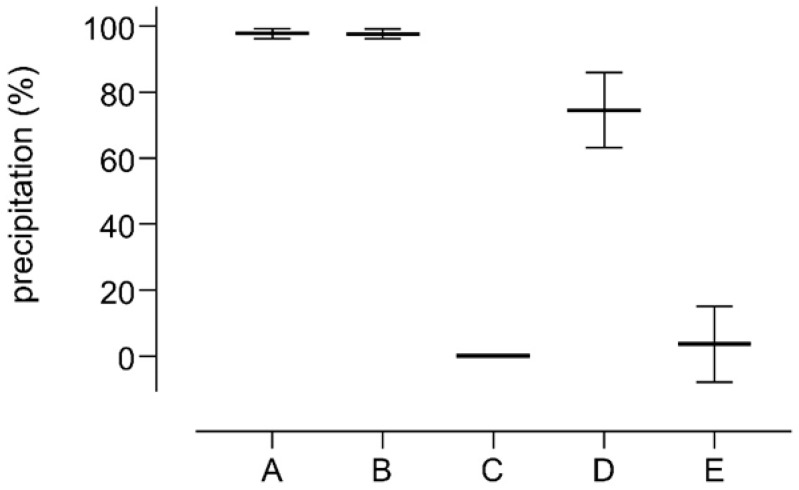
Precipitation yields obtained by mixing two volumes of an HSA solution (10 mg/mL) with two volumes of 10% Perchloric acid (A); eight volumes of acetonitrile (B); two volumes of saturated ammonium sulfate (C); one volume of 10% *w*/*v* zinc sulfate followed by one volume of 0.5 N sodium hydroxide (D); two volumes of 50% *w*/*v* PEG 6000 (E).

Preliminary precipitation experiments were performed by using an aqueous solution of albumin (HSA) treated with different precipitating reagents. Ammonium sulfate or PEG determined little or no precipitation of proteins, Figure 1(C,D), whereas PCA and acetonitrile ensured the best precipitation (98 ± 1% precipitation yield for both reagents, Figure 1(A,B). Interestingly a good but incomplete protein depletion was achievable by using a mixture of zinc sulfate and sodium hydroxide [10,12], which induced a precipitation of 74 ± 4% of proteins Figure 1(D).

Such data are consistent with the relative precipitation efficiencies already reported in the literature [10,11,12,13]. For ammonium sulfate, it has been suggested that the precipitation efficiency can be increased by further dilution of samples [12]; however, this approach was discarded, since it offers no benefits compared to acetonitrile-induced precipitation. Despite zinc sulfate and sodium hydroxide providing incomplete protein precipitation, the optimization of such a protocol was instead considered rather appealing. In fact, protein precipitation occurred with limited sample dilution (i.e., 1:2), and no interference with UV analysis was detectable. Moreover, the supernatants remained aqueous and mildly acidic (pH was between 4 and 6).

Despite such appealing features, the most recent literature reporting such a protocol gives no information on the mechanism triggering protein precipitation, nor on how to adjust the concentration of reagents to improve the precipitation yield [10,12]. This lack of information persisted for a long time, since such a method was reported for the first time in [31]. Interestingly, during preliminary experiments, it was noted that precipitation occurred also in the absence of proteins. On the contrary, no precipitation was detectable in protein samples upon treatment with zinc sulfate only. This led to the hypothesis that the mechanism of protein removal depends on the precipitation of zinc hydroxide, which requires the addition of a basic compound, as follows:

(1) $${Zn}^{2+}+{2OH}^{-}⇄{Zn(OH)}_{2}↓$$

**Figure 2:**
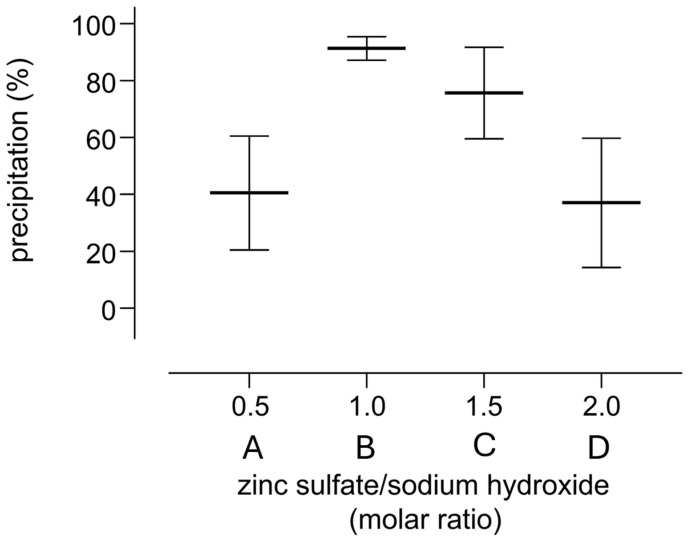
Precipitation yields obtained upon addition of different molar ratios (A. 0.5; B. 1.0; C. 1.5; D. 2.0) of zinc sulfate and sodium hydroxide to a 10 mg/mL solution of HSA.

This hypothesis is consistent with previous findings concerning the formation of insoluble complexes between metal hydroxides and enzymes [32]. To prove such a hypothesis, precipitation experiments were repeated, testing different concentrations of the reagents. The higher precipitation yield (91 ± 4%) was achieved with equimolar amounts of zinc sulfate and sodium hydroxide, whereas either a molar excess or deficiency (Figure 2) of sodium hydroxide resulted in a lower precipitation yield with a higher sample to sample variability.

Since the precipitation of zinc hydroxide should not depend on the type of basic compound used, parallel experiments were also performed with ammonia. As expected, the use of ammonia and sodium hydroxide produced similar precipitation yields.

A possible explanation of the different precipitation yields at different molar ratios can be deduced from the solubility of metal hydroxides calculated in silico [33,34,35]. According to such a model, an insufficient precipitation of zinc hydroxide occurs when a deficiency of base is used (i.e., pH is too low), whereas an excess of base (i.e., pH is too high) can redissolve zinc hydroxide according to the following reaction:

(2) $${Zn(OH)}_{2}↓+{OH}^{-}⇄{Zn(OH)}_{3}^{-}$$

The maximum precipitation for zinc hydroxide was expected to happen at 11 > pH > 8. However, the experimental pH of the supernatants ranged from mildly acidic to mildly basic values (i.e., 6.7 ± 0.2 for samples in Figure 2(A) to 7.9 ± 0.4 for samples in Figure 2(D)), consistent with other experimental data previously reported [12].

Notably, the average precipitation of 75 ± 2% obtained with a 1.5 molar excess of sodium hydroxide (see Figure 2(C)) resembles the data obtained using the protocol reported in the literature (74 ± 4%, see Figure 1(D)), which prescribes the use of reagent concentrations corresponding approximately to a 1.4 molar excess of sodium hydroxide (i.e., zinc sulfate heptahydrate 10% *w*/*v* added with sodium hydroxide 0.5 N) [10,12].

The robustness of the protocol across different conditions was then tested. Specifically, zinc sulfate was replaced with other zinc salts (i.e., chloride, formate) and different basic reagents were used to induce zinc hydroxide precipitation (i.e., ammonia, sodium hydroxide, ammonium or triethylammonium bicarbonate). Moreover, the precipitation was performed on different protein types (i.e., pure albumin, whole serum, tissue homogenate samples) within a 5–100 mg/mL concentration range. Finally, different concentrations of zinc were tested, keeping an equimolar concentration of basic compounds to induce zinc hydroxide precipitation. This molar ratio was chosen because such conditions gave the best precipitation yield and reproducibility in preliminary experiments (see Figure 2).

**Figure 3:**
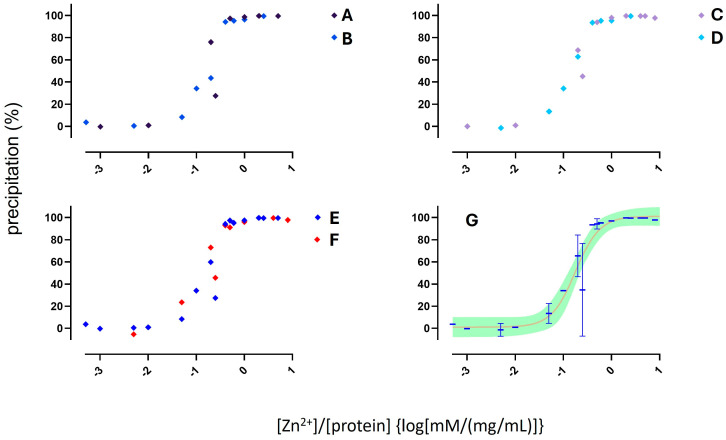
(A vs. B) Effect of the base used to start precipitation of albumin with zinc chloride (A for NaOH, B for NH_3_). (C vs. D) Effect of protein type on precipitation yield obtained with zinc chloride and sodium hydroxide (C for albumin samples, D for serum samples). (E vs. F) Effect of zinc salts on precipitation yield of albumin and serum samples (E for zinc chloride, F for zinc formate). (G) preferred fitting model of whole dataset: nonlinear regression of cumulated data with means and errors and 95% confidence bands.

Data become consistent across different experiments when precipitation yields were plotted as a function of the ratio between the concentration of zinc (mM) and the concentration proteins (in mg/mL) in the samples. As reported in Figure 3, the precipitation yields look like a sigmoidal function of the ratio between zinc and proteins. This finding is consistent with other studies performed on selective precipitation of hemoglobin in organic buffer at pH 8, where a sigmoidal-shaped curve was found as well [36]. However, such publication did not address the mechanistic explanation of which species induced the precipitation of hemoglobin (i.e., zinc or zinc hydroxide), nor what the optimum zinc to buffer ratio was.

Nonlinear regression with extra sum-of-squares F test was used to find the simplest model describing the whole dataset. F test does not reject the null hypothesis that the whole dataset is fitted by a single symmetric sigmoidal function. This implies that different zinc salt added with either ammonia or sodium hydroxide produce the same precipitation yield across samples containing different protein types.

According to the curve reported in Figure 3(G), 0.76 mM is the critical concentration of zinc hydroxide inducing the precipitation of 95% of proteins in a sample loaded with 1 mg/mL of proteins (95P). For experiments performed using pure albumin (MW = 66.5 KDa), such a value is equivalent to a 50-fold molar excess. These data are consistent with another study performed on selective precipitation of hemoglobin. For whole blood hemolysates, it was reported that a tenfold excess of zinc quantitatively precipitates hemoglobin. On the contrary, a 20–25 molar excess of zinc was required for starting the precipitation of other proteins, including albumin [36]. However, such a study did not address whether the precipitation was induced by zinc itself or zinc hydroxide.

Interestingly, protein precipitation with insoluble zinc species was reported also for hexacyanoferrate (II) complexes [37,38,39]. Similarly, aluminum hydroxide was also reported as a protein precipitating reagent [40,41]. However, such methods have been reported for the first time more than one century ago and are still used according to the original protocols with little or no optimization. Since the literature suggests that other metals could be possibly used for protein precipitation, the precipitation efficacy of hydroxides of Fe(III), Cu(II), and Al were also tested. Such metals have been selected because their hydroxides are expected to precipitate at a nearly neutral pH [33,34,35]. As reported by Blais et al., for Fe(OH)_3_, Al(OH)_3_, and Cu(OH)_2_, the -log of solubility product constants are 37.4, 33.5, and 18.6, respectively [42]. Such data have been used by many authors to model the solubility of metal hydroxides. According to Dyer et al., Al(OH)_3_ is expected to have the lowest solubility (0.1 ppb at pH 6) with a solubility below 10 ppb in a pH range between 5 and 9; Fe(OH)_3_ is expected to have a solubility slightly higher than 1 ppb in a pH range between 6 and 9, whereas Cu(OH)_2_ is the most soluble hydroxide with a solubility slightly below 1 ppm in a pH range between 7 and 12 [35]. Outside such pH ranges, the solubility of such hydroxides is also expected to increase rapidly.

The relative concentration of reagents was optimized for each metal, as already described for zinc. All metals were ineffective when an excess or deficiency of base was added, and all of them have a sigmoidal curve similar to Figure 3(G). However, the F test rejected the null hypothesis that the whole dataset is fitted by a single curve. The preferred model was built by four independent sigmoidal curves, each one grouping data obtained by using the same metal as a precipitating regent. Table 1 allows for a comparison between the performance of the different metals as precipitating agents for proteins.

**Figure 4:**
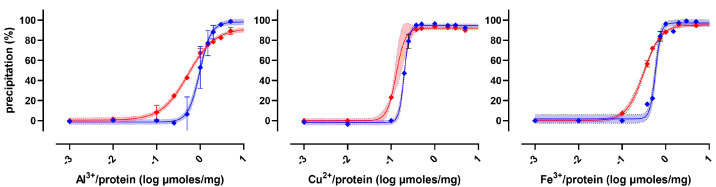
Preferred fitting model of precipitation induced by hydroxides of Fe(III), Cu(II), or Al: separated nonlinear regression with 95% confidence bands for data collected using albumin (blue lines) or serum (red lines).

The different metal hydroxide solubilities are not correlated with the precipitation efficacy. All the metals were quite effective albumin-precipitating reagents, although a complete precipitation (i.e., 95P) occurred at different metal-to-protein concentrations (i.e., different sigmoidal curves for different metals). On the contrary, zinc and ferric hydroxides were the only species providing the quantitative precipitation of serum proteins. In fact, both aluminum and copper hydroxides are not able to provide the precipitation of 95% of serum proteins. Ferric hydroxide is less efficient than zinc hydroxide, since a higher concentration is required to precipitate 95% of albumin, and an even higher concentration is required to precipitate 95% of serum proteins. For experiments performed using hydroxides of Fe(III), Cu(II), or Al, an extra sum-of-squares F test rejected the null hypothesis that a single curve was the best model describing a dataset including samples prepared with albumin or human serum (see Table 1). Unlike for zinc, two separate curves were instead required to fit data collected by using albumin and serum samples (see Figure 4). This implies that such reagents, unlike zinc hydroxide, precipitate albumin and serum proteins in a different manner or with different efficiencies.

**Figure 5:**
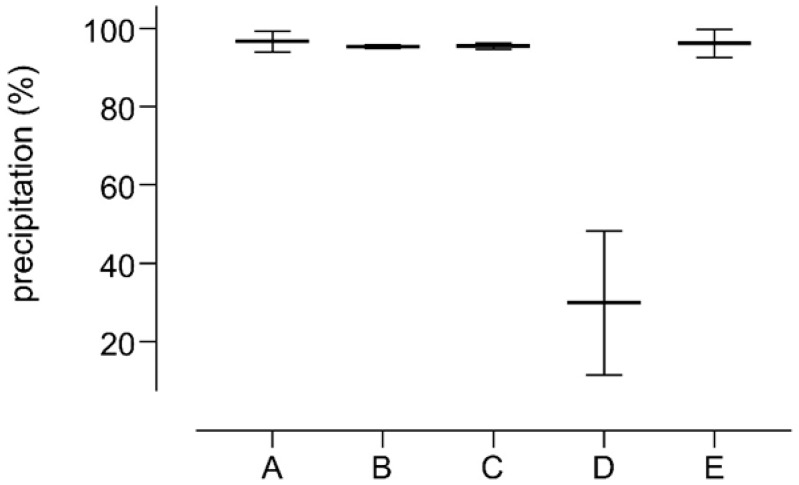
Precipitation yields obtained on samples of 2.5 mg/mL proteins: pure HSA dissolved in 5 mM MES buffer (A); mouse brain homogenate obtained in 5 mM MES buffer (B); mouse liver homogenate obtained in 5 mM MES buffer (C); mouse brain homogenate obtained in 5 mM phosphate buffer (D and E). Samples A–D were treated with 5 mM of zinc chloride, followed by 5 mM NaOH; sample E was treated with 50 mM zinc chloride, followed by 50 mM NaOH.

Since the more robust and consistent results across different samples have been obtained with zinc hydroxides, we performed further tests by using other matrices. As reported in Figure 5, the protein precipitation was also effective for mouse tissue homogenates prepared in MES buffer (see Figure 5(B,C)). However, the procedure was impaired when samples were prepared in phosphate buffer (see Figure 5(D)) and a tenfold excess of zinc hydroxide was required to restore a good precipitation yield (see Figure 5(E)).

Since zinc is added before sodium hydroxide, the precipitation of insoluble zinc phosphate can lead to the exhaustion of the total amount of zinc [43]. Therefore, when sodium hydroxide is then added to the sample, the precipitation of zinc hydroxide and protein is ineffective. On the contrary, the use of a tenfold molar excess of zinc cause the exhaustion of the total amount of phosphate with a residual amount of zinc still sufficient for protein precipitation.

Interestingly, a recent study indirectly supports such a hypothesis. In detail, Cu(II) was found to be the most effective precipitating regent for the characterization of *E. coli* proteome, whereas zinc was the only other metal inducing protein precipitation, although not as efficiently as Cu(II) [44]. Interestingly, the experiments were performed in phosphate buffer at neutral pH. This suggests that Cu(II) can be more compatible with such a buffer, while other metals are even less effective than zinc as protein-precipitating reagents. However, the authors did not investigate the mechanism leading to precipitation of *E. coli* proteins to clarify whether the precipitation was induced by metals or their hydroxides.

**Figure 6:**
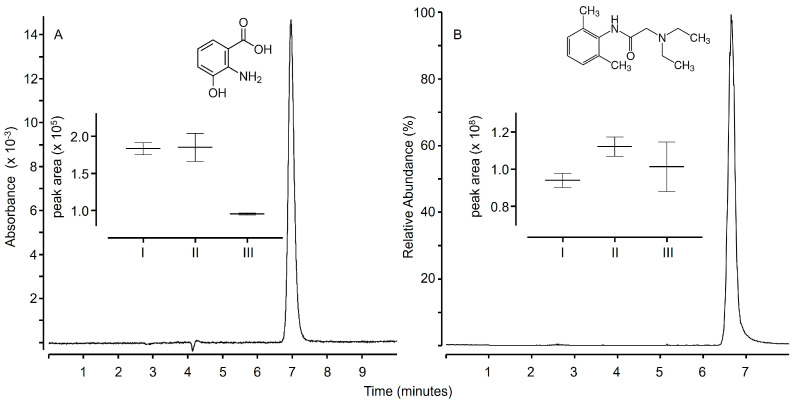
LC-UV chromatogram (single wavelength at 220 nm) of 3HAA (**A**); LC-ESI MS single ion chromatogram (±5 ppm from 235.18049 m/z) of lidocaine (**B**). Peak areas reported in the figure are referred to samples where the compounds were spiked in mobile phase (I), in supernatants of biological samples obtained upon protein precipitation (II), or in biological samples before protein precipitation (III).

Protein precipitation with zinc hydroxide is analytically appealing because it possesses some interesting features. Specifically, the supernatant is transparent in the 200–400 nm UV wavelength range, and samples remain aqueous at nearly neutral pH. These features make samples fully compatible with reversed phase chromatography coupled with UV detection. This was proved by testing zinc hydroxide precipitation on a LC-UV method already reported in the literature [28]. The method was developed for the determination of the activity of the enzyme 3HAO in mouse brain homogenates through the measurement of the residual concentration of 3HAA. As reported in Figure 6A, upon deproteinization with zinc hydroxide, no modification of analyte retention was detected, as well as no matrix effect affecting UV detection. In fact, similar peak areas were observed by spiking 3HHA in solvent (6A-I) or in supernatants obtained upon precipitation of the proteins contained in mouse brain homogenates (6A-II).

Despite the good performance ahead of LC-UV, concerns were about the compatibility with LC-ESI-MS. Specifically, the precipitation of zinc hydroxide is expected to leave residual zinc in the solution [31]. Low micromolar concentration of residual zinc were indeed found by using the ESI-MS method (see Section 3.6). However, such residues seem not to affect the LC-ESI-MS methods, as was proved using a method intended for the measurement of lidocaine in human serum. Chromatographic quantitation of lidocaine in serum is used for multiple applications and was chosen as a test since the compound is stable in human serum and any decrease in peak area upon sample preparation cannot be attributed to enzymatic degradation [45,46]. No matrix effect was detected, since similar peak areas were found for lidocaine standards spiked in solvent (Figure 6B(I)) or in supernatants obtained upon serum protein precipitation (Figure 6B(II)). This suggests that residual zinc does not interfere with LC-ESI-MS analyses of small molecules. Interestingly, potential interferences can be minimized since zinc hydroxide precipitation can be performed by using reagents considered fully compatible with such a technique. Specifically, the same precipitation yield was obtained by using zinc oxide dissolved in formic acid followed by the addition of ammonia to start precipitation. Concerning analyte recovery, different results were obtained for the two methods. Lidocaine had an excellent recovery since samples spiked before protein precipitation (Figure 6B(III)) have 90 ± 6% of the peak area of samples spiked after protein precipitation (Figure 6B(II)). On the contrary, the recovery of 3HAA was 60 ± 8% (see Figure 6A(III and II)), which is less than the recovery obtained by the protocol published (i.e., deproteinization with perchloric acid) [28]. Notably, such an issue does not uniquely affect the precipitation protocol herein reported, since poor recoveries were also detected using other precipitating reagents [13,14]. The different performances of the two methods in terms of recovery are likely to be dependent on the physicochemical properties of the analytes and possibly on their capacity of creating insoluble complexes with metal hydroxides, as reported for proteins, aminoacids, and peptides [32].

## 3. Materials and Methods

### 3.1. Chemicals

Water, HPLC grade (18 MΩ), was purified with a Milli-Q water system (Millipore; Milan; Italy). Perchloric acid was purchased from Riedel de Haën (Honeywell, Milan, Italy). HPLC grade solvents, 3-hydroxyanthranilic acid (3HAA), lidocaine, human serum albumin (HSA), bovine serum albumin (BSA), sodium hydroxide, ammonia, formic acid, N-morpholino-ethanesulfonic acid (MES), ethylenediaminetetraacetic acid (EDTA), and all the other chemicals reported were purchased from Sigma Aldrich (Merck Life Science, Milan, Italy).

### 3.2. Biologic Samples

Animal tissue homogenates for matrix testing were thawed after −80 °C storage. Samples were obtained as reported in a previous study [28]. Human serum was purchased from Sigma Aldrich (Merck Life Science, Milan, Italy), aliquoted, and stored at −20 °C until use.

### 3.3. Protein Samples

Protein samples were prepared by dissolving commercial human albumin in water or appropriate buffers. Protein-rich biological samples were prepared from either commercial human serum or the tissue homogenates reported in Section 3.2. Samples were obtained by diluting biological samples in water or buffer down to the desired protein concentration. Protein concentration of samples was determined spectrophotometrically, as reported in Section 3.5. Commercial human albumin, serum, and tissue homogenates were diluted to obtain 1, 10, and 50 mg/mL solutions.

### 3.4. Protein Precipitation Protocols

Different protein precipitation protocols were tested. Precipitation by means of perchloric acid (PCA) was provided by diluting the sample 1:2 with 5% PCA (*w*/*v*), while precipitation by trichloroacetic acid (TCA) was provided by using 10% TCA (*w*/*v*), as previously reported [10,12,13,28]. Acetonitrile precipitation was provided by the addition of four to nine volumes of acetonitrile (i.e., 1:5 or 1:10 dilution), which was previously identified as sufficient to ensure complete protein precipitation of biological matrices like serum or tissue homogenate [10,12,47,48,49]. Ammonium sulfate precipitation was provided by diluting the sample 1:2 with water saturated with such a chemical, according to previously reported procedures [10,12]. Protein precipitation by PEG or by a mixture of zinc sulfate heptahydrate (10% *w*/*v*) and sodium hydroxide (0.5 N) were performed according to the literature protocols, keeping a final sample dilution of 1:2 [8,9,10,12]. Optimized precipitation procedure with different zinc salts and other metals were performed at a constant volumetric ratio of 2:1:1 (*v*/*v*/*v*; sample: metal solution: basic compound solution), adjusting the concentration of the reagents to provide a wide range of final concentrations, as reported in the Results section. The samples were first added with a solution containing the metal salt and shaken; then, the basic solution was added to start the precipitation.

### 3.5. Measurement of Protein Precipitation Yield

Samples treated with protein precipitating reagents were kept at 4 °C for 10 min and then centrifuged at 14,000× *g* for 10 min at room temperature. Protein concentration in the supernatants was quantified using measurements of UV absorbance at 280 nm, as determined by using a UV-1900 UV-VIS spectrophotometer (Shimadzu, Milan, Italy). Protein concentration was expressed as equivalent of BSA (mg/mL) calculated from a calibration curve freshly prepared by using standards of BSA dissolved in deionized water. Precipitation yield was calculated from the following formula:

(3) $$precipitation\;yield\left(\%\right)=\frac{P}{R}\times 100$$

P being the protein concentration in samples subjected to protein precipitation, and R being the protein concentration in reference samples diluted with water instead of the precipitating agent. The procedure was performed for protein solutions covering concentration levels between 1 and 50 mgmL (1, 10, and 50 mgmL).

### 3.6. Measurement of Zinc Residues

The analyses were performed by using a modified version of previously reported methods [50,51]. Samples were analyzed directly using mass spectrometry upon 1–1 dilution in 100 µM aqueous EDTA (disodium salt) containing 0.1% formic acid. The reference standards for calibration curves were prepared in triplicates at five different concentration levels by diluting zinc chloride in water down to concentrations between 10 µM and 100 µM. The analyses were performed by using an LTQ Orbitrap XL mass spectrometer equipped with an electrospray ionization system (ESI) Finnigan Ion Max 2 and controlled by the software Xcalibur 2.1 (ThermoFisher Scientific, Milan, Italy). ESI was equipped with a stainless-steel capillary (160 µm ID, 140 mm length). Samples were pushed at the flowrate of 5 µL/min into the ESI capillary and nebulized by applying +3.5 kV ionization potential, 30 units of sheath gas, and a capillary temperature of 350 °C. Mass spectra were acquired by the orbitrap in positive ion mode at a resolution of 30,000 (FWHM at m/z 400). Instrument settings were tuned to analyze 5 × 10^5^ ions per scan, in a 150–600 m/z scan range, with 500 milliseconds of maximum time for ion injection into the analyzer, and lock mass option enabled to provide a real-time internal mass calibration. A list of 10 abundant and known background signals was used as reference for internal mass calibration. Background signals were identified as reported in the literature [52].

### 3.7. LC-UV Analyses

The LC-UV analyses were provided as previously described [28]. Briefly, separations were performed at 40 °C by using a Gemini C18 column (250 × 2 mm, 5 µM particle size, 110 Å pore size, Phenomenex, Milan, Italy), equipped on a Surveyor HPLC system and controlled using the software Xcalibur 2.1 (Thermo Fisher Scientific, Milan, Italy). Analyte elution was provided within 7 min by 0.2 mL/min of water containing 10% acetonitrile and HCl to adjust the pH down to 3. Detection was performed at 220 nm using a photodiode array (PDA) detector. Injections were provided with the autosampler, which was programmed to withdraw and inject 10 µL of supernatants collected from protein precipitation experiments.

### 3.8. LC-ESI-MS Analyses

Chromatographic separation was performed by using the same chromatographic apparatus described for LC-UV analysis (see Section 3.7). Elution of the analytes was provided within 10 min by using 0.2 mL/min of water containing 50% acetonitrile and 5 mM ammonium formate. Injections were provided with the autosampler, which was programmed to withdraw and inject 5 µL of supernatants collected from protein precipitation experiments. Detection was performed by using an LTQ Orbitrap XL mass spectrometer. Instrument configuration and mass spectra acquisition were performed as described in Section 3.6, except for ESI nebulization, which was provided by applying +5 kV ionization potential, 40 units of sheath gas, and a capillary temperature of 300 °C.

## 4. Conclusions

Herein, we describe the optimization of a protein precipitation protocol based on zinc hydroxide. The protocol was optimized for the removal of protein interferents from biological matrices to allow for the analysis of small molecules using reversed phase chromatography, ensuring full compatibility with UV and MS detection. The protocol was optimized starting from a method reported in the literature that prescribes the use of 10% *w*/*v* zinc sulfate heptahydrate and sodium hydroxide 0.5 N [10,12]. Despite the fact that such a method was reported for the first time long ago [31], no information on how to adapt the concentration of reagents to optimize the precipitation yield was available because the precipitation mechanism was unknown. Other studies also reported that zinc and other metals can induce protein precipitation without reporting the precipitation mechanism or hints on how to adapt the method to different samples based on protein concentration. For instance, a tenfold molar excess of zinc quantitatively precipitates hemoglobin in whole blood hemolysate samples, while a 20–25 molar excess was required for starting the precipitation of other proteins [36]. Moreover, Cu(II) and Zn were the only metals reported in a proteomic study as effective protein precipitation agents, with Cu(II) overperforming Zn [44].

The optimization of the protocol started with the observation that a basic compound was required to start protein precipitation. The hypothesis that the actual species inducing protein precipitation is the insoluble zinc hydroxide was supported by the literature and experimental data. Notably, all of the literature data reporting zinc as an effective protein precipitating agent were conducted in buffered conditions within a pH range that is suitable for inducing zinc hydroxide precipitation [36,44]. Coprecipitation of proteins with zinc hydroxide occurs according to a sigmoidal trend where the higher the ratio between the concentrations of zinc and proteins, the higher the protein precipitation yield. This finding was also supported by the literature data collected for whole blood hemolysate samples [36]. Protein precipitation with insoluble zinc species is not a brand-new finding. The clarification of samples containing proteins with zinc insoluble complexes was reported more than one century ago for food analysis (i.e., the so-called Carrez clarification method). Such a procedure is based on mixing zinc sulfate and potassium hexacyanoferrate (II) and is still used even for sample preparation before chromatographic separation [37,38,39]. Similarly, a protein precipitation protocol based on insoluble metal hydroxides (i.e., aluminum hydroxide) was reported more than one century ago [40,41]. However, such methods are still used according to the original protocols with little or no optimization. For this reason, we performed several experiments by using metals expected to precipitate as hydroxides within a nearly neutral pH range. This choice was driven by the search for an alternative protein precipitation protocol resulting in an aqueous supernatant having a nearly neutral pH to ensure maximum compatibility with the reversed phase chromatographic method. Zinc hydroxide was found to be the most reliable and efficient precipitating reagent, and the protocol was successfully tested for its compatibility with reversed phase chromatography with both UV and ESI-MS detection.

The protocol was effective for protein removal of different biological samples like human serum and mouse tissues. However, the precipitation method must be carefully evaluated during method development because an incomplete sample recovery was detected for one analyte. Such an issue does not uniquely affect the precipitation protocol herein reported, since poor recoveries were detected also using other precipitating reagents [13,14]. In view of the features herein described, the precipitation of proteins by means of zinc hydroxide can be a valid alternative to protein precipitation by means of strong acidic reagents (e.g., PCA, TCAA) or organic solvents (e.g., acetonitrile, acetone) when such protocols are not applicable because the change in sample media (e.g., pH, polarity) affects the analytical performances of reversed phase chromatography.

## Figures and Tables

**Table 1:** Maximum precipitation yield (MaxP, as % of protein precipitated) obtained with different metal hydroxides (CI is the 95% confidence interval) and minimum metal concentration (mM) required for the precipitation of 95% of the proteins in a 1 mg/mL solution (95P). The F test is passed if one curve fits all data acquired for samples containing either serum or pure albumin.

Reagent	Sample Type				F Test
	Human Albumin		Human Serum		
	MaxP	95P	MaxP	95P	
Zn(OH)_2_	102 (CI 94–112)	0.76	102 (CI 94–112)	0.76	Passed
Al(OH)_3_	98 (CI 94–102)	2.8	91 (CI 86–98)	-	Not passed
Cu(OH)_2_	95 (CI 94–97)	0.47	92 (CI 88–97)	-	Not passed
Fe(OH)_3_	97 (CI 89–107)	1.3	96 (CI 94–99)	2.7	Not passed

## Data Availability

Dataset available on request from the authors.
